# The glocal nature of one health: Linking global principles with local socio-ecological realities

**DOI:** 10.1016/j.onehlt.2026.101404

**Published:** 2026-04-05

**Authors:** R.E. Gozlan

**Affiliations:** ISEM, Univ Montpellier, CNRS, IRD, Montpellier, France

In an increasingly interconnected world, many of the most pressing health threats emerge from interactions between global environmental change and locally embedded socio-ecological systems. The concept of glocalisation was originally developed in the social sciences to describe how global processes are shaped, transformed and implemented through local contexts [1]. Rather than opposing global and local scales, the glocal perspective emphasizes their interdependence: global norms, markets and governance frameworks interact with locally embedded ecological systems, cultural practices and institutional realities. As a result, global principles rarely translate into uniform practices but instead take diverse forms depending on local socio-ecological conditions.

This conceptual lens has been widely applied to fields such as economic globalization, environmental governance and public health. However, it has rarely been explicitly mobilised to frame the One Health paradigm. Yet One Health operates precisely at this intersection between global normative principles and locally embedded health systems. Recognising One Health as a glocal framework may therefore provide a more accurate conceptual foundation for understanding how integrated health approaches can be implemented across diverse ecological and socio-political contexts. Here we argue that One Health is inherently a glocal framework and that recognising this nature provides a clearer conceptual foundation for linking global health governance with locally embedded socio-ecological realities.

## Global values in One Health

At its foundation, the One Health concept is grounded in a set of global normative principles linking human, animal and ecosystem health [2]. These principles recognize that human health, animal health, and ecosystem integrity are intrinsically interconnected and that their protection requires coordinated action across sectors and disciplines. This recognition has gradually emerged from decades of research on zoonotic diseases, ecosystem degradation, and the environmental determinants of health. It reflects a broader shift in scientific and policy thinking: health can no longer be addressed solely within medical or veterinary domains but must be understood within the functioning of complex socio-ecological systems [3].

In this sense, One Health shares important characteristics with other global normative frameworks, such as human rights, biodiversity conservation, and climate governance. These frameworks articulate universal principles that transcend national boundaries and emphasize collective responsibility for shared global goods. Similarly, One Health promotes the idea that preventing disease emergence, safeguarding ecosystem functions, and ensuring sustainable food systems are common objectives that require international cooperation.

Globalization has reinforced the relevance of such principles as health threats increasingly emerge from interconnected human-animal-environment systems across borders [4]. Increasing trade, mobility of people and goods, and rapid environmental change have intensified the interconnectedness of health risks. Pathogens, antimicrobial resistance genes, and environmental contaminants can spread across continents within short time frames, while climate change and land-use transformation reshape the ecological contexts in which diseases emerge. These processes create health risks that are inherently transboundary and cannot be addressed solely through national or sectoral interventions.

However, while the principles underpinning One Health are global in scope, the conditions through which health risks emerge are highly context-dependent. Zoonotic spillover events, food system vulnerabilities, and environmental degradation occur within specific ecological landscapes and social systems. As a result, the universal values embedded in One Health must ultimately be translated into locally adapted governance practices.

## The limits of economic framing

As the One Health concept has gained prominence, considerable efforts have been made to demonstrate its economic benefits [5]. Numerous studies have attempted to quantify the cost-effectiveness of integrated approaches to disease prevention, particularly by comparing the costs of preventive measures with the economic losses associated with disease outbreaks. These analyses have often shown that investments in surveillance, ecosystem protection, and integrated health governance can generate substantial long-term savings by reducing the probability or severity of crises.

While such arguments have been influential in attracting policy attention and funding, they also risk narrowing the conceptual scope of One Health [6]. Framing One Health primarily as an economically efficient strategy may inadvertently reduce it to a technocratic optimisation tool, an issue also highlighted by critiques emerging from the Blue Marble Health perspective which question the dominant global narratives surrounding health inequalities and disease risk [7]. Economic metrics tend to prioritise interventions that can be easily quantified and compared, potentially overlooking social, ecological, and cultural dimensions that are more difficult to express in monetary terms.

Moreover, economic evaluations often rely on generalized models that assume comparable costs and benefits across regions. In reality, however, the socio-ecological contexts in which health risks emerge vary widely across the world. The drivers of zoonotic disease emergence, for example, may include wildlife trade, agricultural intensification, deforestation, or urban expansion, but their relative importance differs substantially between regions and societies. Policies that appear economically efficient in one context may therefore prove ineffective or even counterproductive in another.

A narrow economic framing may also obscure the normative foundations of One Health. Protecting ecosystems, safeguarding animal welfare, and ensuring equitable access to health are not solely matters of cost-benefit calculations but also reflect ethical commitments regarding the stewardship of shared planetary resources. Reducing One Health to an economic argument may thus weaken its normative force and limit its capacity to inspire systemic transformation.

For these reasons, a broader conceptual perspective is required, one that recognises both the universal values underpinning One Health and the locally embedded conditions through which health risks arise. This perspective is captured by the notion of a glocal approach to health governance.

## One Health as a glocal framework

The glocal perspective emphasizes that global processes are always mediated by local ecological, social, and institutional contexts. Rather than treating global and local scales as separate or opposing domains, the glocal approach highlights their continuous interaction and mutual influence.

In the context of health governance, this perspective is particularly relevant. The drivers of health risks (including climate change, biodiversity loss, agricultural intensification, and global trade) operate at global or regional scales. Yet the processes through which these drivers translate into concrete health outcomes occur within specific landscapes, communities, and production systems. Zoonotic spillover events, for instance, emerge from localized interactions between wildlife, livestock, humans, and their shared environments, although their distribution at the global scale reflects broader socio-ecological drivers such as land-use change, population density and biodiversity gradients [8]. Similarly, the spread of antimicrobial resistance often reflects local patterns of antibiotic use in human medicine, veterinary practice, and agriculture.

Global scientific knowledge and international policy frameworks remain essential for guiding action and facilitating cooperation. However, effective implementation requires translating these principles into locally appropriate strategies that reflect cultural practices, environmental conditions, and institutional capacities. This glocal perspective also highlights the importance of cross-scale interactions in shaping health outcomes. Decisions made at global or national levels (such as trade policies, agricultural subsidies, or environmental regulations) can profoundly influence local socio-ecological systems. Conversely, local practices and innovations can inform broader policy frameworks by demonstrating effective approaches to integrated health governance.

Understanding One Health as a glocal framework thus reframes the concept as a dynamic interface between global knowledge and local action. It underscores the need for governance systems capable of connecting scientific research, community-based management, and policy decision-making across multiple scales. Operationalising a local focus in One Health requires moving beyond generic adaptation towards co-constructed, place-based interventions that reflect locally embedded ecological knowledge, cultural practices and institutional realities. This includes strengthening community-based surveillance and ensuring that local knowledge systems and resources are not exposed without appropriate safeguards, such as consent and equitable benefit-sharing. In this perspective, effective implementation relies on adaptive, locally grounded strategies rather than standardised global solutions.

## Examples of glocal health systems

Several well-documented disease systems illustrate how health risks emerge from the interaction between global drivers and locally embedded socio-ecological systems, providing concrete examples of the glocal nature of One Health challenges. These examples highlight the relevance of a glocal perspective in understanding and managing complex health challenges.

One example is the emergence of Nipah virus in Southeast Asia. The initial outbreaks were associated with rapid changes in land use, including deforestation and agricultural intensification, which altered interactions between fruit bats, livestock, and human populations. Global market dynamics and agricultural expansion created new ecological interfaces where viral spillover became more likely. Yet the specific mechanisms of disease emergence depended on local farming practices, wildlife ecology, and community behaviours. Effective interventions therefore required locally adapted solutions, including modifications to livestock management, surveillance systems, and community awareness programmes. This case illustrates how globally driven processes such as agricultural expansion and market integration can interact with locally specific ecological interfaces to produce zoonotic spillover, a dynamic that exemplifies the fundamentally glocal nature of emerging health risks.

Another example concerns the spread of antimicrobial resistance in food production systems. The global trade of livestock products and pharmaceuticals contributes to the worldwide dissemination of resistant bacteria and genes. However, patterns of antibiotic use and regulatory enforcement vary widely between regions. In many settings, the emergence and spread of resistance are strongly shaped by local agricultural practices, access to veterinary services, and governance capacities. Addressing antimicrobial resistance therefore requires both international coordination and locally tailored strategies that reflect specific production systems. Antimicrobial resistance reflects a glocal dynamic in which globally circulating pathogens and pharmaceuticals interact with locally specific production systems, regulatory frameworks and veterinary practices.

Vector-borne diseases such as Rift Valley fever provide a further illustration of glocal dynamics. Climate variability, global climate change, and regional weather patterns influence the distribution and abundance of mosquito vectors. Yet outbreaks often occur in specific landscapes where environmental conditions, livestock movements, and local livelihoods create favourable conditions for transmission. Disease prevention strategies must therefore integrate global climate monitoring with local surveillance and risk management. This illustrates how global climatic processes translate into locally differentiated disease dynamics, reinforcing the need for health governance frameworks capable of operating across both global and local scales.

These examples demonstrate that health risks cannot be fully understood or addressed at a single scale. Instead, effective responses depend on recognising how global drivers interact with local ecological and social systems.

## Why the future of One Health lies in glocal governance

Recognising the glocal nature of One Health has important implications for how health governance should be designed and implemented. Many current policy frameworks still attempt to operationalise One Health through top-down global strategies, standardised surveillance systems or uniform policy templates. While global coordination is essential to address transboundary health risks, such approaches often overlook the ecological and social heterogeneity through which these risks actually emerge. Health threats at the human-animal-environment interface are shaped by locally specific combinations of land use, food production systems, cultural practices, and institutional capacities. As a result, effective One Health governance cannot be imposed solely through global frameworks but must be built through locally embedded institutions capable of integrating ecological knowledge, public health systems and community practices. In this perspective, global governance should provide shared principles, coordination and knowledge exchange, while local actors adapt these principles to their socio-ecological realities. The future of One Health therefore lies not in universal policy prescriptions but in glocal governance systems capable of translating global principles into locally grounded strategies that reflect ecological realities, cultural practices and institutional diversity.

## CRediT authorship contribution statement

**R.E. Gozlan:** Writing – review & editing, Writing – original draft, Conceptualization.

## Declaration of competing interest

I declare that I have no competing interests.

## Data Availability

No data was used for the research described in the article.
